# The impact of squamous cell transformation on the prognosis of patients treated with radical nephroureterectomy

**DOI:** 10.1186/s12885-024-12010-5

**Published:** 2024-02-22

**Authors:** Li-Hua Huang, Chuan-Shu Chen, Jian-Ri Li, Kun-Yuan Chiu, Shian-Shiang Wang, Cheng-Kuang Yang, Chen-Li Cheng, Chi-Chien Lin, Yen-Chuan Ou

**Affiliations:** 1 Department of Urology, Tungs’ Taichung MetroHarbor Hospital, Taichung, Taiwan; 2 Department of Urology, Taichung Veterans General Hospital, Taichung, Taiwan; 3 Doctoral Program in Translational Medicine, National Chung Hsing University, Taichung, Taiwan; 4 Rong Hsing Translational Medicine Research Center, National Chung Hsing University, Taichung, Taiwan; 5 Department of Medical Research, Taichung Veterans General Hospital, Taichung, Taiwan; 6 Department of Post-Baccalaureate Medicine, College of Medicine, National Chung Hsing University, Taichung, Taiwan; 7 The iEGG and Animal Biotechnology Center, Advanced Plant and Food Crop Biotechnology Center, National Chung-Hsing University, Taichung, Taiwan; 8 Department of Pharmacology, College of Medicine, Kaohsiung Medical University, Kaohsiung, Taiwan; 9 Department of Medical Research, China Medical University Hospital, Taichung, Taiwan

**Keywords:** Carcinoma, Squamous cell, Transitional cell, Nephroureterectomy

## Abstract

**Background:**

Limited information is available for guiding the management of upper urinary tract (UUT) urothelial carcinoma with squamous differentiation (UC-SqD). We did not even know about the difference between pure urothelial carcinoma (UC) and UC-SqD in the UUT regardless of treatment policy and prognosis. Instead of direct comparisons against each other, we included the third UUT malignancy, squamous cell carcinoma (SCC). This three-way-race model allows us to more clearly demonstrate the impact of squamous cell transformation on patient outcomes in UUT malignancy.

**Methods:**

We retrospectively analysed 327 patients with UC, UC-SqD, or SCC who underwent radical nephroureterectomy with bladder cuff excision (RNU) at Taichung Veterans General Hospital, Taichung, Taiwan, between January 2006 and December 2013. A Kaplan–Meier survival analysis was used to evaluate the relationship between patient outcomes and histology. Multivariate Cox proportional hazards modelling was also used to predict patient prognoses.

**Results:**

The five-year postoperative cancer-specific survival (CSS) rates were 83.6% (UC), 74.4% (UC-SqD), and 55.6% (SCC), and the 5-year recurrence-free survival (RFS) rates were 87.7% (UC), 61.5% (UC-SqD), and 51.9% (SCC). UC patients had significantly better 5-year RFS than UC-SqD and SCC patients (*P* = 0.001 and *P* < 0.0001, respectively). Patients with pure UC had significantly better 5-year CSS than SCC patients (*P* = 0.0045). SCC or UC-SqD did not independently predict disease-specific mortality (HR 0.999, *p* = 0.999; HR 0.775, *p* = 0.632, respectively) or disease recurrence compared to pure UC (HR 2.934, *p* = 0.239; HR 1.422, *p* = 0.525, respectively). Age, lymphovascular invasion (LVI), and lymph node (LN) status independently predicted CSS, while pathological tumour stage, LN status, and LVI predicted RFS.

**Conclusions:**

SCC and UC-SqD are not independent predictors of survival outcomes in patients with UUT tumours. However, they are associated with other worse prognostic factors. Hence, different treatments are needed for these two conditions, especially for SCC.

## Background

Urothelial carcinoma (UC) in the upper urinary tract (UUT) is rare, with an incidence of ~ 2 cases per 100,000 person-years in Western countries [1]. Upper urinary tract urothelial carcinoma (UUT-UC) has rare histological variants that are associated with worse oncological outcomes. UC with squamous differentiation (UC-SqD) is the most common, accounting for 41% of all histological variants [2]. Squamous cell carcinoma (SCC) in UUTs is also rare. Unfortunately, few articles (mostly case reports) discuss rare malignancies. Berz et al. reported the most extensive series, which showed a ratio of 1.35% in UUT neoplasms and a poorer prognosis than UC [3].

The above two UUT malignancies, UC-SqD and SCC, share common histological characteristics. Intercellular bridges or keratinization are essential for diagnosis. A urothelial component is the main difference between UC-SqD and SCC. Because of the lack of experience in treating UC-SqD and SCC, we mostly followed the same treatment guidelines as those for UC. Squamous components in UC predict a poor prognosis. The poor prognosis may be due to the wrong type of treatment, the fact that squamous cancer itself is a more serious condition, or both. Hence, we hypothesized that poorer outcomes would correspond to the presence of more squamous cell components. Consequently, SCC may have a worse prognosis than UC-SqD and pure UC. Although several studies have examined bladder SCC or UC-SqD, research involving the UUT is limited. This is the first study to compare outcomes among patients with UC, UC-SqD, and SCC in the UUT.

## Methods

### Patients and study design

The institutional review board approved the study. We identified 373 patients who underwent radical nephroureterectomy with bladder cuff excision (RNU) for UUT tumours between January 2006 and December 2013 from the Taichung Veterans General Hospital database. Only patients with UC, UC-SqD, or SCC were enrolled. We excluded patients with other histological variants, including sarcomatoid (*n* = 4), glandular (*n* = 1), poor (*n* = 1), nested (*n* = 1), plasmacytoid (*n* = 1), micropapillary (*n* = 1), or other (*n* = 1) variants. Patients with another UUT malignancy, including adenocarcinoma (*n* = 1), undifferentiated (*n* = 1), small cell carcinoma (*n* = 3), or renal cell carcinoma + UC (*n* = 2), were excluded. We also excluded patients who received neoadjuvant chemotherapy (*n* = 3), immediate postoperative intravesical instillation therapy (*n* = 14), or concomitant or previous cystectomy (*n* = 13). The data of the remaining 327 patients are presented.

The patients’ clinical and pathological data were retrospectively reviewed. When patients had concomitant bladder cancer, they underwent transurethral resection of the bladder tumour before RNU. Urologists performed all the RNU procedures using either an open or laparoscopic approach. Regional lymphadenectomy was performed if preoperative imaging showed suspicious lymph node (LN) metastasis. Extended LN dissection was not performed routinely.

### Pathological evaluation

The tumours were staged according to the American Joint Committee on Cancer (AJCC) cancer staging manual. Tumour grading was assessed according to the World Health Organization/International Society of Urologic Pathology consensus classification established in 1973. Squamous cell carcinoma was defined as a pure histologic lesion in the specimen; UC-SqD was defined as a mixed urothelial and squamous malignancy. The tumour volume was roughly estimated using the formula $(4\pi/3)\times\frac{\mathrm{length}}2\times\frac{\mathrm{width}}2\times\frac{\mathrm{height}}2$.

### Postoperative evaluation

After RNU, patients were followed regularly as outpatients. The follow-up included medical history, physical examination, serum creatinine level, urine analysis, cytology, cystourethroscopy, chest X-ray, and abdominal computer tomography.

Cancer-specific survival (CSS) was defined as the time from the day of RNU to the time of death due to a UUT tumour. The cause of death was determined by chart review or telephone interview. Recurrence-free survival (RFS) was defined as the time from the day of RNU to tumour recurrence and was defined as the time at which a tumour was detected in the operative field, regional LNs, or distant metastases. Tumour relapse in the bladder or contralateral UUT was not considered tumour recurrence.

### Statistical analysis

Differences in patient characteristics were also analysed. Categorical variables were analysed using Fisher’s exact test and the Chi-square test. Continuous variables were assessed by the Mann–Whitney U test (two categories) or Kruskal–Wallis test (three categories).

The Kaplan‒Meier method was used to calculate the CSS and RFS. Differences were compared using the log-rank test. Prognostic factors related to CSS and RFS were analysed with Cox proportional hazards regression models for univariate and multivariate analysis. *P* < 0.05 was considered indicative of statistical significance.

## Results

Overall, 327 patients who underwent RNU were enrolled in this study. Pathologically, 294 patients had UC (90%), 24 had UC-SqD (7.3%), and 9 had SCC (2.7%). Table 1 shows the patients’ descriptive characteristics. There were no significant differences in age, sex, smoking history, uraemia status, or number of positive LNs. Significantly, SCC patients had the lowest BMI but the largest tumour volume (UC vs. UC-SqD vs. SCC: 2.6 cm^3^ vs. 13.6 cm^3^ vs. 250.8 cm^3^). SCC patients were more likely to have flank pain (23.1% vs. 45.8% vs. 77.8%), advanced T stage (> T2: 50.7% vs. 87.5% vs. 100%) and positive surgical margins (3.7% vs. 12.5% vs. 22.2%). Pure UC patients were more likely to experience gross haematuria and had the least negative prognostic factors (T stage, N stage, LVI, and positive surgical margins). UC-SqD patients were more likely to receive postoperative adjuvant chemotherapy.

**Table 1:** Summary of patients’ demographic and pathological characteristics

Variable		1. UC (*n*=294)		2. UC-SqD (*n*=24)		3. SCC (*n*=9)		total (*n*=327)		*P-value*	*P-value (1,2)*	*P-value (1,3)*	*P-value (2,3)*
		median	IQR	median	IQR	median	IQR	median	IQR				
Age		67.20	57.4, 74.6	65.40	57.5, 75.4	71.30	67.1, 76.7	67.20	57.4, 74.8	0.595^c^	0.947^d^	0.306^d^	0.431^d^
BMI		23.70	21.5, 25.9	22.30	20.6, 24.3	22.20	21.0, 23.1	23.60	21.5, 25.7	0.039^c^	0.094^d^	0.046^d^	0.479^d^
Tumor volume		2.60	0.8, 11.7	13.60	3.3, 45.6	250.80	163.8, 299.5	3.50	1.0, 15.6	<0.0001^c^	0.001^d^	<0.0001^d^	0.001^d^
		Count	Column N %	Count	Column N %	Count	Column N %	Count	Column N %				
Gender	male	129	(43.9 %)	12	(50.0 %)	5	(55.6 %)	146	(44.6 %)	0.668^b^	0.562^a^	0.515^b^	1.000^b^
	female	165	(56.1 %)	12	(50.0 %)	4	(44.4 %)	181	(55.4 %)				
Gross hematuria	no	89	(30.3 %)	15	(62.5 %)	8	(88.9 %)	112	(34.3 %)	<0.0001^a^	0.001^a^	0.001^b^	0.217^b^
	yes	205	(69.7 %)	9	(37.5 %)	1	(11.1 %)	215	(65.7 %)				
Flank pain	no	226	(76.9 %)	13	(54.2 %)	2	(22.2 %)	241	(73.7 %)	<0.0001^a^	0.013^a^	0.001^b^	0.134^b^
	yes	68	(23.1 %)	11	(45.8 %)	7	(77.8 %)	86	(26.3 %)				
Smoking history	no	220	(74.8 %)	18	(75.0 %)	6	(66.7 %)	244	(74.6 %)	0.857^a^	0.985^a^	0.698^b^	0.677^b^
	yes	74	(25.2 %)	6	(25.0 %)	3	(33.3 %)	83	(25.4 %)				
Uremia	no	255	(86.7 %)	22	(91.7 %)	8	(88.9 %)	285	(87.2 %)	0.903^b^	0.752^b^	1.000^b^	1.000^b^
	yes	39	(13.3 %)	2	(8.3 %)	1	(11.1 %)	42	(12.8 %)				
Pathological T	≦T2	145	(49.3 %)	3	(12.5 %)	0	(0.0 %)	148	(45.3 %)	<0.0001^b^	0.001^a^	0.004^b^	0.545^b^
	>T2	149	(50.7 %)	21	(87.5 %)	9	(100.0 %)	179	(54.7 %)				
Pathological N	N0 or Nx	270	(91.8 %)	20	(83.3 %)	7	(77.8 %)	297	(90.8 %)	0.107^b^	0.248^b^	0.175^b^	1.000^b^
	N+	24	(8.2 %)	4	(16.7 %)	2	(22.2 %)	30	(9.2 %)				
LVI	no	242	(82.3 %)	15	(62.5 %)	7	(77.8 %)	264	(80.7 %)	0.051^b^	0.028^b^	0.664^b^	0.681^b^
	yes	52	(17.7 %)	9	(37.5 %)	2	(22.2 %)	63	(19.3 %)				
Surgical margin	no	283	(96.3 %)	21	(87.5 %)	7	(77.8 %)	311	(95.1 %)	0.015^b^	0.079^b^	0.052^b^	0.597^b^
	yes	11	(3.7 %)	3	(12.5 %)	2	(22.2 %)	16	(4.9 %)				
Adjuvant chemotherapy	no	227	(77.2 %)	8	(33.3 %)	4	(44.4 %)	239	(73.1 %)	<0.0001^a^	<0.0001^a^	0.038^b^	0.690^b^
	yes	67	(22.8 %)	16	(66.7 %)	5	(55.6 %)	88	(26.9 %)				

^a^ Pearson chi-square

^b^ Fisher's exact test

^c^ Kruskal-Wallis H test

^d^ Mann-Whitney U test

The median follow-up times were 63.8 (IQR 31.2, 89.5), 55.4 (IQR 23.4, 75.7), and 32.1 months (IQR 7.0, 68.1) for patients with UC, UC-SqD, and SCC, respectively. During the follow-up, 47 (16%), 7 (29.2%), and 4 (44.4%) patients with UC, UC-SqD, and SCC, respectively, died of their disease.

**Fig. 1:**
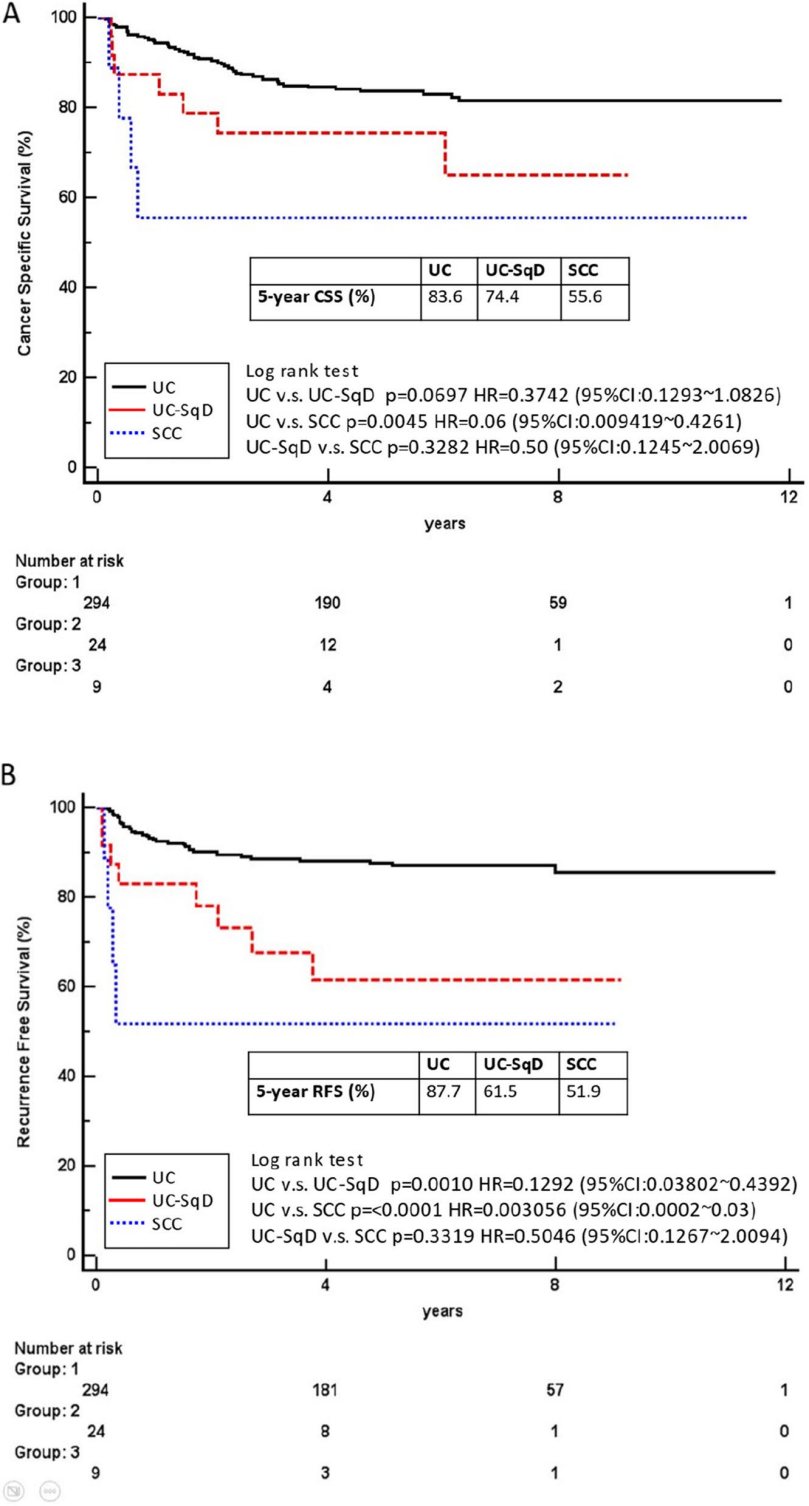
K‒M plots for CSS (log-rank *p* = 0.006, **A**) and RFS (log-rank *p* < 0.0001, **B**) by histology type

Figure 1 shows the Kaplan–Meier plots for RFS and CSS estimates stratified by pure UC versus UC-SqD versus SCC. The five-year postoperative CSS rates for patients with pure UC, UC-SqD, and SCC were 83.6%, 74.4%, and 55.6%, respectively; and the RFS rates were 87.7%, 61.5%, and 51.9%, respectively. We performed pairwise comparisons. The survival curves for RFS were significantly different between UC patients and SCC patients (log-rank test, *p* < 0.001) and between UC patients and UC-SqD patients (log-rank test, *p* = 0.0010). There was a significant difference in CSS between UC and SCC patients (log-rank test, *p* = 0.0045).

According to our multivariate regression analyses controlling for clinicopathological variables, compared to pure UC, neither SCC nor UC-SqD was an independent predictor of disease-specific mortality (HR 1.0, *p* = 0.999; HR 0.78, *p* = 0.632, respectively) or disease recurrence (HR 2.93, *p* = 0.239; HR 1.42, *p* = 0.525, respectively). Age, LVI, and LN status independently predicted CSS. Pathological tumour stage, LN status, and LVI independently predicted RFS (Table 2).

**Table 2:** Univariate and multivariate Cox regression analyses of RFS and CSS

	Recurrence free survival						Cancer specific survival					
	univariate			multivariate			univariate			multivariate		
Variable	HR	95%CI	*P* value	HR	95% CI	*p*-value	HR	95%CI	*P* value	HR	95% CI	*p*-value
Tumor type												
UC	referent			referent			referent			referent		
UC-SqD	3.34	1.55~7.21	0.0020	1.42	0.48~4.21	0.5250	2.05	0.92~4.53	0.0780	0.78	0.27~2.20	0.6320
SCC	7.13	2.53~20.15	0.0002	2.93	0.49~17.59	0.2390	3.91	1.41~10.87	0.0090	1.0	0.17~5.76	0.9990
Age	1.01	0.99~1.04	0.4040				1.04	1.01~1.06	0.0050	1.03	1.00~1.05	0.0280
BMI	0.91	0.83~1.00	0.0360	1.02	0.91~1.13	0.7870	0.91	0.84~1.00	0.0280	0.94	0.85~1.03	0.1810
Tumor volume	1.01	1.00~1.01	<0.0001	1.00	1.00~1.01	0.3240	1.01	1.00~1.01	0.0010	1.00	1.00~1.01	0.6710
Gross hematuria	0.59	0.33~1.05	0.0750				0.69	0.41~1.16	0.1610			
Flank pain	1.87	1.04~3.38	0.0370	1.13	0.50~2.55	0.7680	2.24	1.33~3.77	0.0020	1.88	0.96~3.68	0.0670
Smoking	1.30	0.70~2.44	0.4070				1.06	0.59~1.91	0.8420			
Uremia	1.06	0.45~2.51	0.8870				1.16	0.55~2.44	0.7000			
pathologic stage												
≦T2	referent			referent			referent			referent		
>T2	8.38	3.31~21.20	<0.0001	4.89	1.37~17.47	0.0150	4.64	2.35~9.18	<0.0001	2.00	0.84~4.80	0.1190
LN stage												
pN0/Nx	referent			referent			referent			referent		
pN+	7.52	3.99~14.21	<0.0001	4.12	1.78~9.57	0.0010	9.36	5.40~16.23	<0.0001	6.50	3.22~13.11	<0.001
LVI	5.45	3.07~9.67	<0.0001	2.29	1.10~4.76	0.0270	4.59	2.73~7.72	<0.0001	2.20	1.14~4.26	0.0190
Surgical margin (+)	5.65	2.64~12.12	<0.0001	1.88	0.68~5.25	0.2260	3.61	1.71~7.62	0.0010	1.12	0.43~2.96	0.8140
Adjuvant chemotherapy	2.85	1.60~5.06	0.0004	0.61	0.28~1.34	0.2210	s2.03	1.20~3.42	0.0080	0.63	0.32~1.24	0.1820

## Discussion

The cohort study revealed that 6.4% of the UC-SqD patients and 2.4% of the SCC patients underwent RNU for UUT tumours. The incidence of UC-SqD in UUT tumours was 6.7–16%, and that of SCC was 1.4–8% [3–7]. Our results are compatible with previous reports.

Several articles have compared the prognosis of SCC or UC-SqD to that of UC in the bladder [8–12]. In contrast, UUT studies were limited to small series and case reports, likely since SCC and UC-SqD are rare in the UUT. Holmang et al. reported worse CSS in patients with SCC than in patients with UC in the UUT [5]. Our study design is similar to that of Holmang et al.; however, we included a UC-SqD patient group. This model allows us to demonstrate the effect of malignant squamous cell components on patient prognosis.

Clinically, the differential diagnosis of UC-SqD from pure UC or SCC depends on whether mixed urothelial and squamous elements are identified. Since urothelial or squamous components in UC-SqD may be unrecognizable via microscopy, UC-SqD may be erroneously diagnosed as SCC or UC. The misdiagnosis could occur secondary to borderline histological features, limited biopsy specimens, tiny secondary histology, or histologic artefacts secondary to crush or cautery artefacts [13]. In the present study, most SCC and UC-SqD patients were misdiagnosed before RNU. All patients with UC-SqD were erroneously classified as having pure UC at ureteroscopy biopsy, while 56% (5/9) of the SCC patients were initially misdiagnosed with pure UC, RCC, or complicated renal cysts. In contrast, the recognition rate of UC-SqD at transurethral bladder resection (TURBT) for bladder cancer was greater than 75% [14]. The better recognition rate is due to adequate specimen sampling during TURBT. In some cases, recognizing subtle squamous differentiation by haematoxylin and eosin staining is impossible. Hence, several articles have investigated the role of immunohistochemistry in distinguishing UC-SqD from UC and SCC [15–18]. Squamous differentiation may occur beyond the histological level since immunohistochemistry (IHC) markers of the squamous lineage, such as CK14 and MAC387, can be detected in morphologically pure UC [13, 19].

**Fig. 2:**
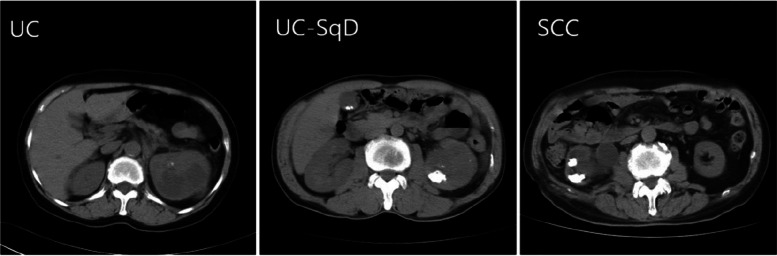
Various calcification patterns in UUT tumours.  Concomitant calcification formation is seldom found in pure UC (only 8.2%); in contrast, the rates of calcification formation in UC-SqD and SCC patients were greater (25.0% and 56.6%, respectively); in addition, calcification tends to be decreased in UC patients, while it becomes increasingly greater in UC-SqD and SCC patients

The development of UC-SqD and SCC in UUT is controversial, and most of the results were obtained from studies of bladder disease. UC bladder cells may convert into SCC cells and proliferate and progress with time, causing mixed urothelial and squamous cell carcinomas (like UC-SqD) to ultimately develop into pure SCC [20]. UC, UC-SqD, and SCC are diseases with different stages and severities. Other studies have reported that SCC develops from keratinizing squamous metaplasia and dysplasia of urothelium cells, which are secondary to long-term Foley catheter use, chronic irritation, urinary tract calculi, and Schistosoma haematobium infection [21]. Therefore, urinary tract calculi are potential risk factors for squamous metaplasia in UUT patients. In this study, the rates of calculi formation were greater in SCC and UC-SqD patients than in pure UC patients (56.6% vs. 25.0% vs. 8.2%, respectively), according to computed tomography (CT). In addition, the calculi patterns differed among the three diseases. These lesions appeared as “independent stones” in SCC and UC-SqD patients and as “small calcifications” in pure UC patients (Fig. 2). Such differences in CT scans may help in differential diagnosis.

From our perspective, SCC is a different disease than UC and UC-SqD. This conclusion is based on three observations. First, our study showed that the 5-year tumour recurrence rates in the bladder and contralateral UUT were similar between UC patients and UC-SqD patients (32.1%:29.6% and 13.7%:14.9%, respectively). In contrast, there were no cases of recurrent SCC during follow-up, regardless of bladder or UUT recurrence. This phenomenon in SCC may be related to its short survival period; however, there is much more to this phenomenon. We believe that pure SCC results from metaplasia and dysplasia of urothelium cells instead of 100% squamous transformation from UC-SqD. Because of the lack of UC in SCC patients, we could not observe any recurrence in the bladder or contralateral UUT. Second, SCC patients had no other synchronous genitourinary tract tumours. All nine SCC patients had primary kidney tumours, and two had tumours that directly invaded the upper ureter. In contrast, 26.2% (77/294) of UC patients and 16.7% (4/24) of UC-SqD patients had multiple foci of tumours. Multifocality is a common finding in UC but not in SCC. Third, metastases in UC-SqD patients always feature urothelial tissue components. In SCC, the metastatic tissue is always squamous. This finding appears to support the theory that SCC develops from squamous metaplasia instead of from UC-SqD.

SCC could be a different disease than UC-SqD and UC. These findings also support the findings of a previous study on the structural genetics of bladder cancer: UC and UC-SqD have similar genetic alterations, and UC-SqD develops from UC [22]. In contrast, SCC is a separate tumour group since it has a lower frequency of polysomy and genetic alterations than UC and UC-SqD. Since SCC demonstrates different clinical behaviours than UC and UC-SqD, a distinct treatment and follow-up strategy may be applied. Unlike follow-up plans for UC, scheduled cystoscopy and urinary tract imaging may not be needed for metachronous tumour detection in UUT-SCC patients.

In our study, squamous transformation was associated with poor prognosis in patients with UUT tumours. However, UC-SqD and SCC were not found to be independent risk factors after adjustment for other investigated variables. We found a much stronger association between survival and other factors, such as LN metastasis, LVI and age (Table 2). This result may indicate that squamous cell transformation, LN metastasis, LVI and age are not independent of each other. This also means that patients with UC-SqD or SCC have a more advanced cancer status than patients with UC at the time of diagnosis, naturally resulting in a poorer prognosis. This explanation is also consistent with the results in Table 1, where we found that the proportion of haematuria was significantly greater in patients with pure UC than in patients with UC-SqD and SCC. This warning sign puts pure UC in a favourable position for early diagnosis. In addition, the incidence of flank pain in UC-SqD and SCC patients was significantly greater than that in pure UC patients, which also suggests that UC-SqD and SCC patients are often diagnosed at a later stage of cancer when the growth of the tumour causes flank pain. It is reasonable to assume that with early diagnosis, the prognosis for patients with UC-SqD and SCC would be no worse than that for patients with UC.

Treatment policies for UC-SqD and SCC in UUTs are poorly established, and most related information arises from case sharing. In general, management of UC-SqD is similar to that of UC, and perioperative chemotherapy is suggested for patients with advanced cancer. In UC-SqD patients, neoadjuvant or adjuvant chemotherapy provides therapeutic effects comparable to those of UC [2, 23, 24]. In contrast, SCC is less sensitive to chemotherapy than UC [25–28]. The National Comprehensive Cancer Network (NCCN) guidelines state that chemotherapy has no effect on bladder SCC. Nevertheless, long-term survival or complete remission from SCC is possible. We applied adjuvant chemotherapy to five SCC patients. The survival results were similar to those of UC-SqD and UC patients, indicating that chemotherapy is reasonable for SCC. The other four SCC patients who did not receive chemotherapy died within 6 months after radical surgery. Overall, UC-SqD and even SCC may be responsive to modern immunotherapy. In the PURE-01 study, 86% (6/7) of bladder cancer patients with predominant squamous differentiation (defined as involving > 50% of the tumour specimens) had downstaged to pT ≤ 1 after three courses of neoadjuvant pembrolizumab [29].

There are several limitations to the present study. First, our study was retrospective and therefore featured bias in patient selection and treatment options. Second, the sample sizes of UC-SqD and SCC patients were relatively small, limiting the statistical power. Third, RNUs for patients in this study occurred over a seven-year period and were performed by different operators, who might have varied in their surgical expertise and learning curve. Fourth, there was no central pathological review of the specimens. The 2016 WHO classification system indicates that the percentage of histological variants in UC should be described [30]. This study did not define the percentages of squamous differentiation. We hypothesized that the degree of UC-related squamous cell transformation has prognostic importance for patient outcomes. However, further studies with adequate patient numbers and pathologic information are needed.

## Conclusion

Neither SCC nor UC-SqD is an independent predictor of outcomes in patients with UUT tumours. Nonetheless, the above two conditions are associated with a worse prognosis than pure UC since patients initially present with a more advanced tumour status. The disease course of UC-SqD is similar to that of pure UC; in contrast, SCC has unique tumour behaviour. We believe that the tumour biology of SCC differs from that of UC and UC-SqD.

## Data Availability

The datasets used and/or analysed during the current study were obtained from the corresponding author upon reasonable request.

## Abbreviations

UUT: Upper urinary tract
UC-SqD: Urothelial carcinoma with squamous differentiation
UC: Urothelial carcinoma
SCC: Squamous cell carcinoma
RNU: Radical nephroureterectomy with bladder cuff excision
CSS: Cancer-specific survival
RFS: Recurrence-free survival
LVI: Lymphovascular invasion
LN: Lymph mode
UUT-UC: Upper urinary tract urothelial carcinoma
TURB: Transurethral resection of bladder tumour
CT: Computed tomography
